# Supplementary material for: Live-cell single-molecule fluorescence microscopy for protruding organelles reveals regulatory mechanisms of MYO7A-driven cargo transport in stereocilia of inner ear hair cells
Source: bioRxiv. 2024 May 7:2024.05.04.590649. Preprint. [Version 1] doi: 10.1101/2024.05.04.590649 (PMC11100596; doi:10.1101/2024.05.04.590649)
Supplement: 1 [file NIHPP2024.05.04.590649V1-supplement-1.pdf]

bioRxiv preprint doi: https://doi.org/10.1101/2024.05.04.590649; this version posted May 7, 2024. The copyright holder for this preprint (which was not certified by peer review) is the author/funder, who has granted bioRxiv a license to display the preprint in perpetuity. It is made available under aCC-BY 4.0 International license.

Supplemental Figures

Fig. S1: Processive movements of MYO7A-HMM dimers in stereocilia

Representative kymograms of HaloTag-MYO7A-HMM-FKBP in cells treated with 200 nM AP20187. Trajectories of slow movements (**a**) and rapid movements (**b**) are shown (arrows). Single-plane time-lapse, every 1 s. Bars, 2 µm and 20 s.

Fig. S2: Movements MYO10-MD anchored to the plasma membrane of stereocilia

a, Velocities of MYO10 molecules moving in stereocilia before and after the 500 nM AP21987 treatment. The mean velocity was 2.01 ± 0.37 µm/s (n = 12, mean ± standard deviation) before dimerization and 0.72 ± 0.34 µm/s (n = 23) after dimerization. **b**, Representative kymograms of membrane-anchored MYO10-MD molecules. Continuous trajectories are consistent with processive and directional movements (arrows). Retrograde movements are also observed (arrowheads). Single-plane time-lapse, every 100 ms. Bars, 2 µm and 6 s.

bioRxiv preprint doi: https://doi.org/10.1101/2024.05.04.590649; this version posted May 7, 2024. The copyright holder for this preprint (which was not certified by peer review) is the author/funder, who has granted bioRxiv a license to display the preprint in perpetuity. It is made available under aCC-BY 4.0 International license.

Supplemental information

Movie S1: Time-lapse images of HaloTag-actin

Vestibular hair cell (P2) expressing HaloTag-actin was imaged as a control for proteins stably bound to the F-actin core. Most of the fluorescent puncta remain in the same location and disappear suddenly due to photobleaching or transition to the dark state (representatively indicated by magenta circles). JFX554, 0.01 nM. Single-plane time-lapse, every 1 s. Exposure, 100 ms at 0.2 kW/cm^2. Bar, 5 µm.

Movie S2: Time-lapse images of non-fused HaloTag

Vestibular hair cell (P2) expressing non-fused HaloTag was imaged as a control for diffusing proteins. Most fluorescent puncta disappear after one frame. JFX554, 0.1 nM. Single-plane time-lapse, every 1 s. Exposure, 100 ms at 0.2 kW/cm^2. Bar, 5 µm.

Movie S3: Time-lapse images of HaloTag-MYO7A-HMM-FKBP with the AP20187 treatment

Vestibular hair cell (P2) expressing HaloTag-MYO7A-HMM-FKBP was imaged after adding 200 nM AP20187 to the culture medium. Molecules showing directional movements are indicated by magenta circles. JFX554, 0.3 nM. Single-plane time-lapse, every 1 s. Exposure, 100 ms at 0.2 kW/cm^2. Bar, 5 µm.

Movie S4: Time-lapse images of HaloTag-MYO7A-HMM-FKBP without AP20187 treatment

Vestibular hair cell (P2) expressing HaloTag-MYO7A-HMM-FKBP was imaged without adding AP21087 to the culture medium. No directional movements are observed. JFX554, 0.3 nM. Single-plane time-lapse, every 1 s. Exposure, 100 ms at 0.2 kW/cm^2. Bar, 5 µm.

Movie S5: Time-lapse images of HaloTag-MYO7A-RK/AA

Vestibular hair cell (P2) expressing HaloTag-MYO7A-RK/AA, which has two missense mutations (p.R2127A and p.K2130A) disabling autoinhibition of the motor domain, was imaged every 1 s by single-plane time-lapse acquisition. Molecules showing directional movements are indicated by magenta circles. JFX554, 0.3 nM. Exposure, 100 ms at 0.2 kW/cm^2. Bar, 5 µm.

Movie S6: Time-lapse images of HaloTag-MYO7A-ΔSH3-ΔM/F2

Vestibular hair cell (P2) expressing HaloTag-MYO7A-ΔSH3-ΔM/F2, whose tail is truncated to disable autoinhibition of the motor domain, was imaged every 1 s by single-plane time-lapse

bioRxiv preprint doi: https://doi.org/10.1101/2024.05.04.590649; this version posted May 7, 2024. The copyright holder for this preprint (which was not certified by peer review) is the author/funder, who has granted bioRxiv a license to display the preprint in perpetuity. It is made available under aCC-BY 4.0 International license.

acquisition. A molecule showing directional movements is indicated by magenta circles. JFX554, 0.3 nM. Exposure, 100 ms at 0.2 kW/cm^2. Bar, 5 µm.

Movie S7: Time-lapse images of membrane-anchored HaloTag-MYO7A-HMM-FRB

Vestibular hair cell (P2) co-expressing HaloTag-MYO7A-HMM-FRB and IL2Rα-EGFP-FKBP. The cell is treated with 500 nM AP21987 to anchor MYO7A-HMM to the plasma membrane. Molecules showing stepwise, directional movements toward stereocilia tips are indicated by magenta circles. Single-plane time-lapse, every 300 ms. JFX554, 0.3 nM. Exposure, 100 ms at 0.2 kW/cm^2. Bar, 5 µm.

Movie S8: Time-lapse images of HaloTag-MYO10-MD-FRB before membrane anchoring

Vestibular hair cell (P2) co-expressing HaloTag-MYO10-MD-FRB and IL2Rα-EGFP-FKBP. The cell is imaged without the AP21987 treatment. A small number of molecules show rapid directional movements toward stereocilia tips (magenta circles). Single-plane images are acquired every 100 ms. JFX554, 0.3 nM. Exposure, 100 ms at 0.2 kW/cm^2. Bar, 5 µm.

Movie S9: Time-lapse images of HaloTag-MYO10-MD-FRB after membrane anchoring

Vestibular hair cell (P2) co-expressing HaloTag-MYO10-MD-FRB and IL2Rα-EGFP-FKBP. The cell is treated with 500 nM AP21987 to anchor MYO10-MD to the plasma membrane. Molecules showing processive movements are indicated by magenta circles. Single-plane images are acquired every 100 ms. JFX554, 0.3 nM. Exposure, 100 ms at 0.2 kW/cm^2. Bar, 5 µm.

Movie S10: Time-lapse images of HaloTag-MYO7A-HMM-FKBP tethered to F-actin

Vestibular hair cell (P2) co-expressing HaloTag-MYO7A-HMM-FKBP and FRB-PST-EGFP. The cell is treated with 500 nM AP21987 to bind the C-terminus of MYO7A-HMM to F-actin. A small number of molecules show stepwise, directional movements toward stereocilia tips (magenta circles). Single-plane time-lapse, every 1 s. JFX554, 0.3 nM. Exposure, 100 ms at 0.2 kW/cm^2. Bar, 5 µm.

Movie S11: Time-lapse images of HaloTag-MYO10-MF-FKBP tethered to F-actin

Vestibular hair cell (P2) co-expressing HaloTag-MYO10-MD-FKBP and FRB-PST-EGFP. The cell is treated with 500 nM AP21987 to bind the C-terminus of MYO10-MD to F-actin. Molecules start stepwise, directional movements toward stereocilia tips (magenta circles). Single-plane time-lapse, every 100 ms. JFX554, 0.3 nM. Exposure, 100 ms at 0.2 kW/cm^2. Bar, 5 µm.

Fig. S1

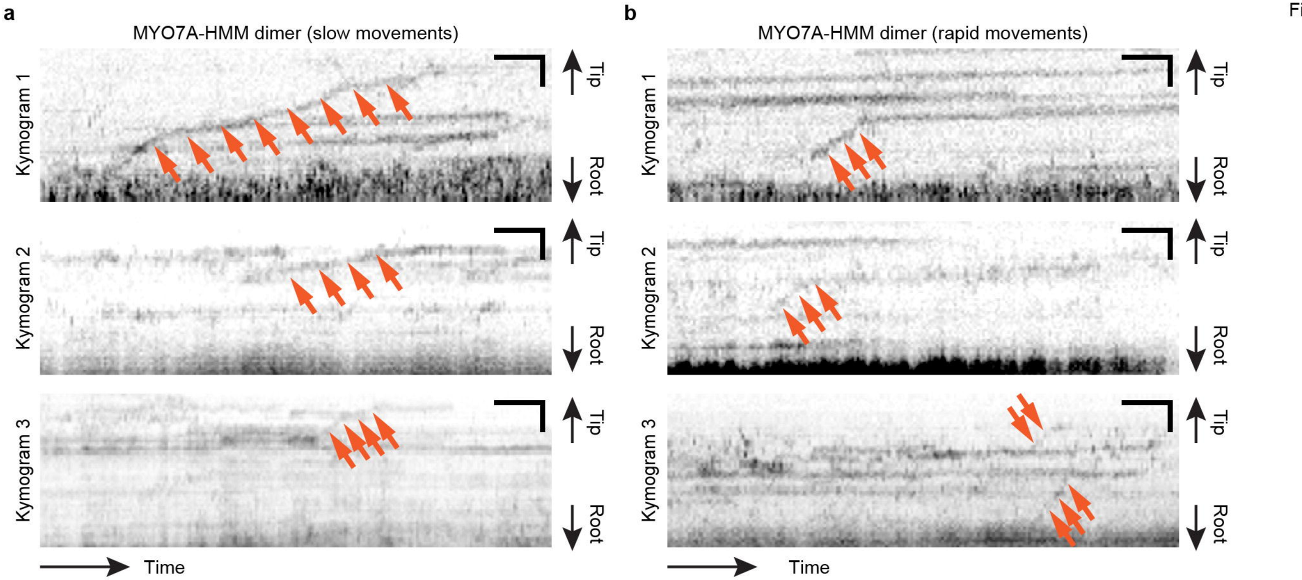

a
MYO7A-HMM dimer (slow movements)
b
MYO7A-HMM dimer (rapid movements)
Kymogram 1
Kymogram 2
Kymogram 3
Tip
Root
Time

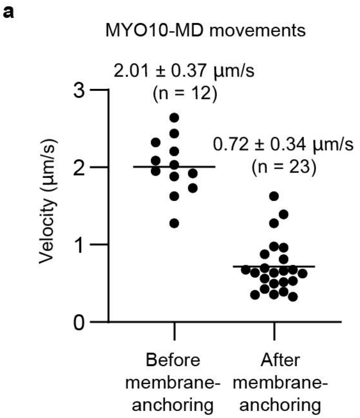
a
MYO10-MD movements
2.01 ± 0.37 µm/s
(n = 12)
0.72 ± 0.34 µm/s
(n = 23)
Velocity (µm/s)
3
2
1
0
Before membrane-anchoring
After membrane-anchoring

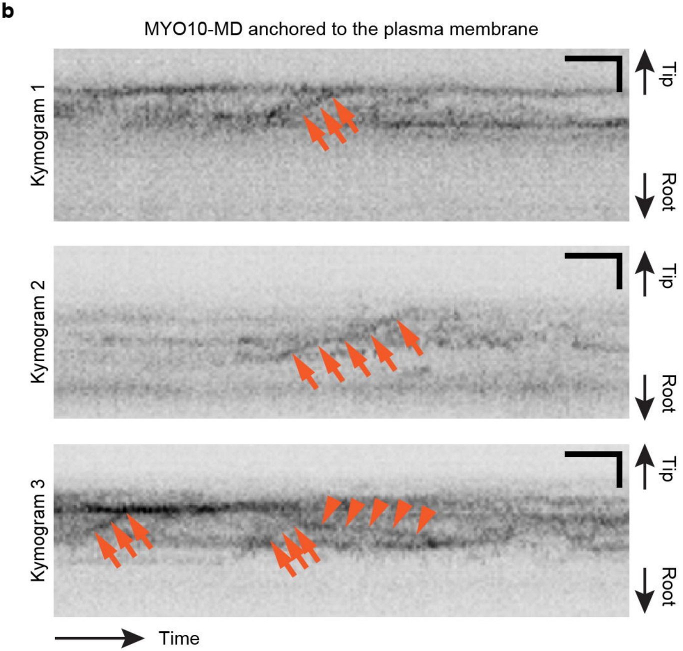
b
MYO10-MD anchored to the plasma membrane
Kymogram 1
Kymogram 2
Kymogram 3
Tip
Root
Time
